# Discovery of spatial pattern of prickles on stem of *Rosa hybrida* ‘Red Queen’ and mathematical model of the pattern

**DOI:** 10.1038/s41598-021-93133-8

**Published:** 2021-07-05

**Authors:** Kazuaki Amikura, Hiroshi Ito, Miho S. Kitazawa

**Affiliations:** 1 Department of Molecular Biophysics and Biochemistry, Yale University, New Haven, USA; 2 Department of Computational Biology and Medical Sciences, Graduate School of Frontier Sciences, The University of Tokyo, Kashiwa, Japan; 3 Department of Art and Information Design, Faculty of Design, Kyushu University, Fukuoka, Japan; 4 Center for Education in Liberal Arts and Sciences, Osaka University, Toyonaka, Japan; 5 Department of Biological Sciences, Graduate School of Science, Osaka University, Toyonaka, Japan; 6 Present Address: Department of Interdisciplinary Space Science, Institute of Space and Astronautical Science, Japan Aerospace Exploration Agency, Sagamihara, Japan

**Keywords:** Developmental biology, Plant sciences, Computer modelling

## Abstract

The developmental patterns of many organisms are orchestrated by the diffusion of factors. Here, we report a novel pattern on plant stems that appears to be controlled by inhibitor diffusion. Prickles on rose stems appear to be randomly distributed, but we deciphered spatial patterns of prickles on *Rosa hybrida* cv. ‘Red Queen’ stem. The prickles primarily emerged at 90 to 135 degrees from the spiral phyllotaxis that connected leaf primordia. We proposed a simple mathematical model that explained the emergence of the spatial patterns and reproduced the prickle density distribution on rose stems. We confirmed the model can reproduce the observed prickle patterning on stems of other plant species using other model parameters. These results indicated that the spatial patterns of prickles on stems of different plant species are organized by similar systems. Rose cultivation by humans has a long history. However, prickle development is still unclear and this is the first report of prickle spatial pattern with a mathematical model. Comprehensive analysis of the spatial pattern, genome, and metabolomics of other plant species may lead to novel insights for prickle development.

## Introduction

The heterogeneous distribution of factors, such as a simple gradient of signaling molecules or periodic patterns resulting from reaction-diffusion systems, is crucial for spatiotemporal pattern formation in multicellular organisms^1,2^. For example, diffusion and active transport of the plant hormone auxin has a central role during pattern formation in plants^3^. The regular arrangement of leaves around a stem, called phyllotaxis, is based on the active transport of auxin^4^. Phyllotaxis in *Arabidopsis thaliana* is dependent on polar auxin transport^5^, and computer models have established that the dynamics of auxin efflux carriers modulating auxin concentration is sufficient to reproduce the phyllotactic patterns^6,7^. Leaf venation patterning also is explained by polar auxin transport^8^. The intercellular movement of small RNAs (possibly via plasmodesmata microchannels traversing cell walls) also contributes to adaxial-abaxial leaf patterning and root vascular patterning^9^. Peptide signals such as CLV3 diffuse through multicellular tissues and contribute to the maintenance of the shoot apical meristem structure^10^. Thus, the intercellular movement of factors is a fundamental phenomenon driving pattern formation in plants.

This study investigated the spatial patterning of prickles on plant stems. Prickles are spiny structures derived from epidermal tissue on the plant body^11,12^. Anatomical studies using scanning electron microscopy (SEM) indicated that glandular trichomes, which are derived from epidermal tissue, develop into prickles in several Rosaceae species including a *Rosa hybrida* cultivar^13^. A previous SEM study reported that the glandular trichomes have structural differences in the *Rubus* species^14^. Comparison of those two genomes could provide insights into prickle development, which has not yet been fully described at the genome or molecular level. A recent study proposed a candidate gene that controls prickle density on *Rosa chinensis* stem^15^. This candidate gene displayed strong similarity to *TTG2* in *A. thaliana*, which is involved in trichome development^16^. This result suggests conserved gene function between rose prickles and *A. thaliana* trichomes. Cell-cell signaling and phytohormones (especially jasmonates) are responsible for initiating trichome development^17^. Several MYB transcription factors negatively regulate trichome initiation and move directly between cells via plasmodesmata, suggesting a pattern formation mechanism similar to the Turing activator-inhibitor system^18^. However, the genes driving prickle emergence have not been identified^19,20^. Previous studies only measured the number of prickles on stems. There are no statistical and mathematical analyses of prickle spatial patterns despite the long history of rose cultivation and appreciation.

This study reports that prickle spatial patterns on rose stem depend on leaf position. We developed a simple mathematical model of prickle patterning based on a diffusive inhibitor secreted by leaves. To our knowledge, this is the first report describing the statistical and mathematical modeling of prickle patterning on plant stems. This study provides novel data to elucidate the molecular mechanism of prickle development.

## Results

### Prickle spatial patterns

**Figure 1:**
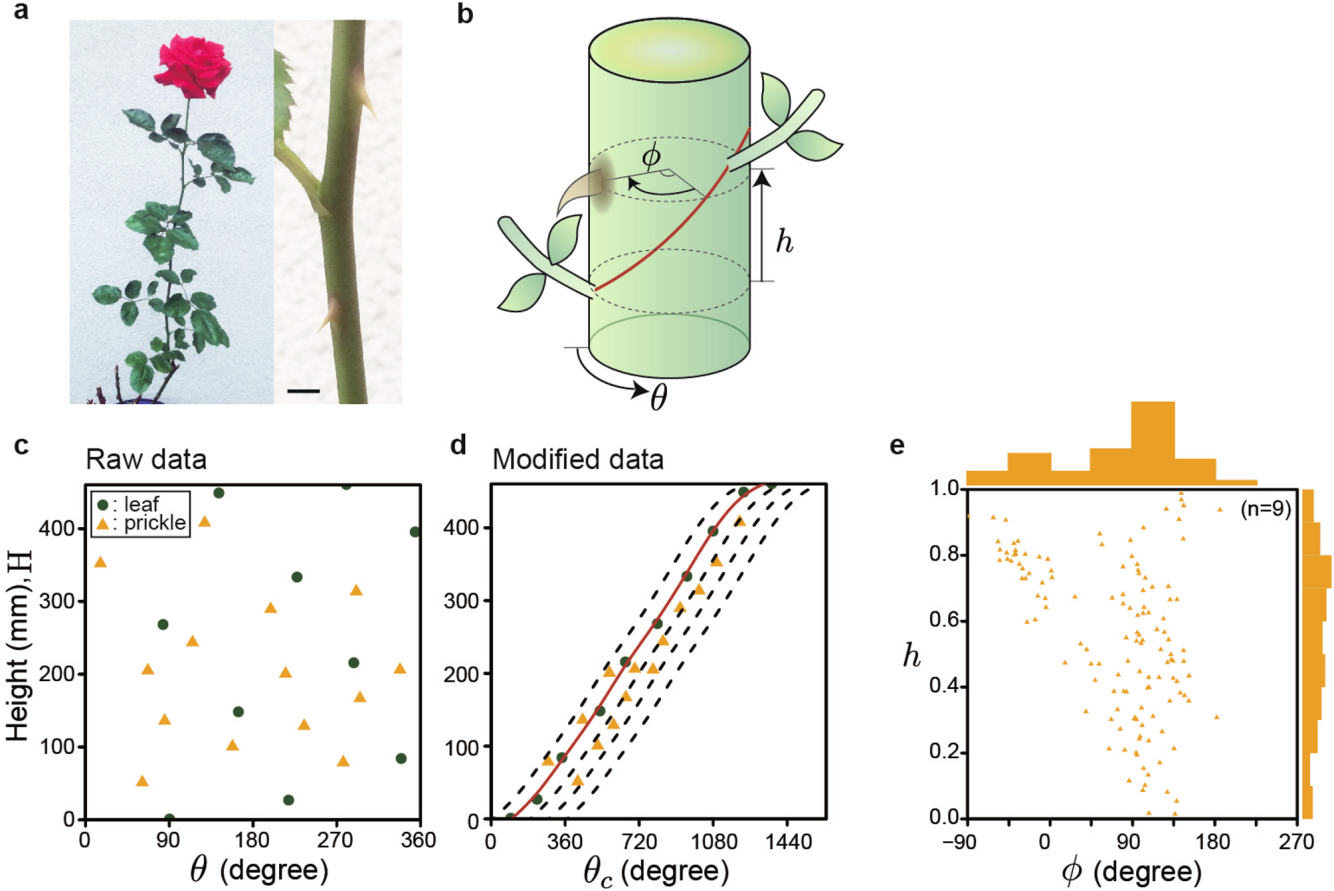
Prickle pattern on rose stems. (**a**) Complete view and prickles of *R. hybrida* ‘Red queen’. The scale bar is 5 mm. (**b**) Definition of $\theta$, $\phi$, and *h*. $\theta$ is the measured angle of leaf or prickle. $\phi$ is the angle between prickle and the spline curve at the same height on the stem. Let *h* be defined as the relative height of the prickle between adjacent leaves, where the length between two leaves is 1. (**c**) Positions of leaves and prickles on the stem of a sample. Green circle, leaf; yellow triangle, prickle. (**d**) Modified positions of leaves and prickles on the stem of the same sample to (**c**). The red line represents the spline curve connecting leaves. The dotted lines were drawn by adding $-90^{\circ }$, $90^{\circ }$, $180^{\circ }$, or $270^{\circ }$ to the spline curve. (**e**) Scatter plot of $\phi$ and *h* profiles from measured samples (n=9). The histogram represents the distribution of $\phi$ or *h* from measured samples.

*R. hybrida* ‘Red Queen’ displayed spiral phyllotaxis with a few prickles between each node (Fig. 1a). The average divergence angle between leaves was close to the golden angle $\Phi$ (Supplementary Fig. 1). Transient deviation from the constant spiral often was found, with the divergence angle exceeding $270^{\circ }$ in extreme case. The angle and height of prickles and leaves were modified to analyze the prickle pattern (see Methods). Measurements of prickle positions expressed in $\theta _c-H$ planes displayed two tendencies: concentration of prickles at $\phi \sim 90^{\circ }$ and occasional occurrence of a prickle underneath the leaf in $-90^{\circ }< \phi < 0^{\circ }$ (Fig. 1d, Supplementary Fig. 2). These tendencies were confirmed by the frequency distribution expressed in the $\phi -h$ plane (Fig. 1e). We discovered that the prickle frequently occurs in the range of $\phi$ between $90^{\circ }$ and $135^{\circ }$ at any *h*, which corresponds to the first tendency. The occurrence of prickles with negative $\phi$ ($-90^{\circ }< \phi < 0^{\circ }$) was limited to the region where $h>0.6$. There were essentially no prickles in other stem areas. These results indicated that prickle position was not random, but displayed a pattern relative to leaf position.

### Mathematical modeling of prickle spatial patterning on rose stem

**Figure 2:**
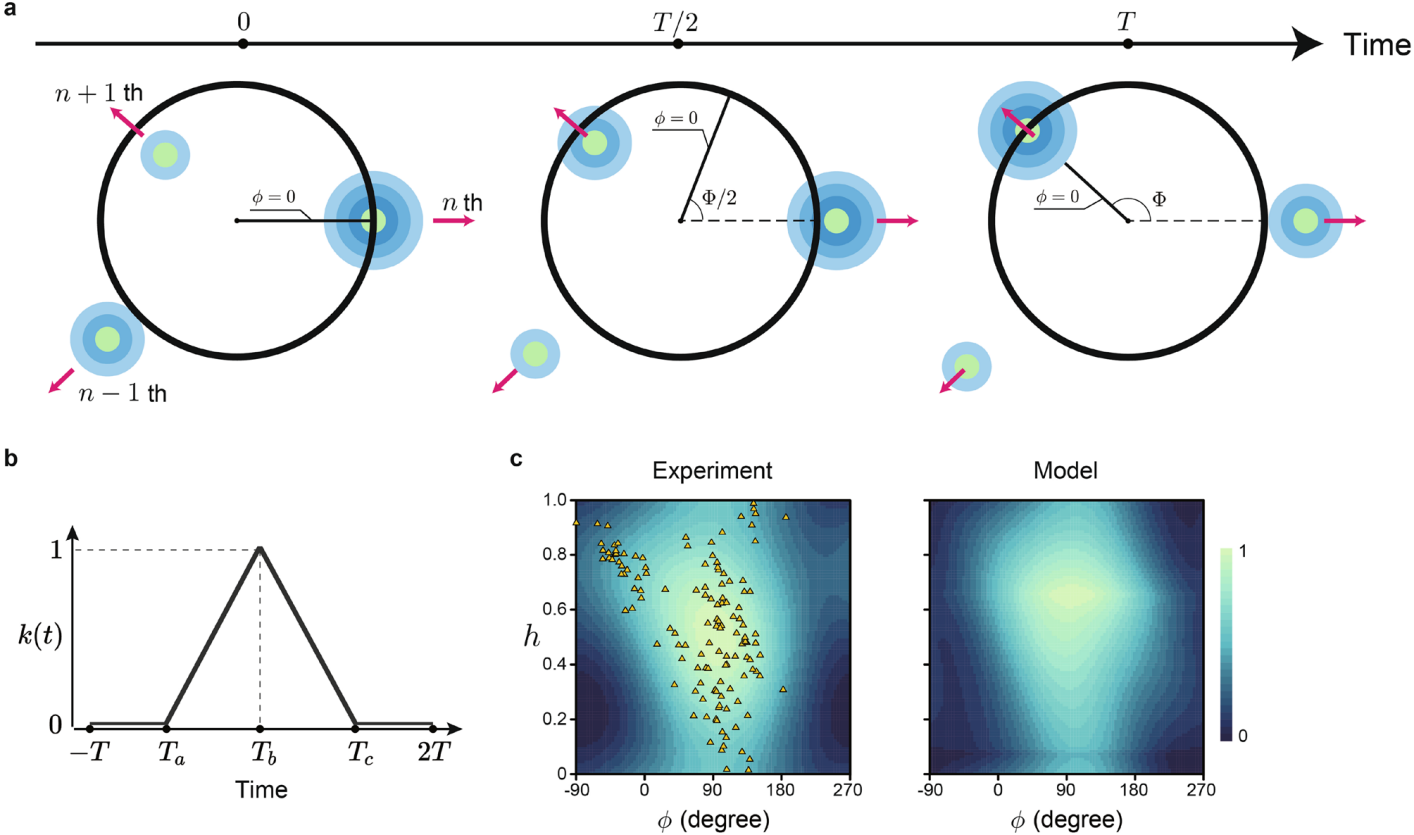
Model of prickle development. (**a**) Schematic diagram of the distribution of inhibitor produced by leaf primordia during prickle development. Primordia periodically emerged with angle $\Phi$ around the shoot apex (center of the black circle). We assumed that each primordium produced an inhibitor regulating prickle emergence, which diffused in the surface layer of the stem at a constant rate. Priming zone where prickles can be produced is assumed as a ring at a distance from the top of meristem (black circle), and the probability of prickle emergence on the circle is proportional to the reciprocal of inhibitor concentration. The position on the circle is specified by $\phi$, where $\phi = 0$ (the solid line from the center to the periphery) corresponds to the spline curve connecting leaves (red lines in Fig. 1b,d). The amount of inhibitor secretion follows *k*(*T*). (**b**) Schematic plot of the time-dependent production rate *k*(*t*) (see Methods). (**c**) Prickle density on the $\phi$-*h* plane estimated from real data (left) and resulted from computer simulation (right). Left panel shows prickle density estimated using kernel density estimation and real data (orange triangles, Fig. 1e). Right panel shows computer simulation of prickle density using a set of optimized parameters at $\alpha =0.267$, $\beta =0.139$, $T_a=-0.909$, $T_b=0.071$ and $T_c=1.661$.

The fact that prickles emerge with a specific angle to leaves suggests that leaf primordia may be involved in regulating prickle development and growth. We performed computer simulations to examine whether an inhibitory effect of leaf primordia sufficiently reproduced the observed prickle patterning. The simulations used a simple model of a plane centered on the shoot apex (see Methods and Fig. 2). We assumed that the emergence of prickles could be inhibited by the diffusive factors secreted from the leaf primordia, which emerge around the shoot apical meristem (SAM) with a constant divergence angle $\Phi$ at every time interval *T* (Fig. 2a). Leaf primordia were moved centrifugally at constant velocity, mimicking relative displacement from the shoot apex due to the tip growth. The priming zone of prickles was set below the SAM. Prickle growth occurs in a wide region along the stem^13^, and we consistently observed various prickle sizes. In this study, however, we observed only the mature prickles over 5 mm in height. We hypothesized that prickles were initiated at a specific distance from the shoot apex. Previous study supported the prickle initiation in the early stage of shoot development^21^. Under this assumption, the priming zone of prickles was expressed as a circle in a 2D plane, where the center of the circle corresponds to the shoot apex. Based on these assumptions, we employed five parameters in our model. The inhibitor secretion occurs between $T_a$ and $T_c$ (Fig. 2b). The amount of inhibitor secretion is maximized at $T_b$ (Fig. 2b). Simple linear function for showing the magnitude of secretion uses those three parameters (see Methods). The constant velocity is parameterized as $\alpha$ and $\beta$(see Methods). By optimizing the model parameters, $\alpha , \beta , T_a, T_b,$ and $T_c$, the model qualitatively reproduced the observed prickle distribution on the $\phi -h$ plane which peaks around $90^{\circ }$. We repeated the optimization procedure with $10^4$ initial parameter sets, and the parameters converged to either of two distinct parameter sets. Fig. 2c was drawn based on the set of parameters that produces the distribution of *f* with the highest correlation to real data. The other set of parameters produced a similar distribution of *f* (Supplementary Fig. 3). We evaluated the sensitivity of prickle patterning to the parameters related to inhibitor diffusion (Supplementary Fig. 4a). This prickle pattern was relatively robust to changes in inhibitor concentration if diffusion was similar (Supplementary Fig. 4b,c). We found that concave-shaped inhibitor diffusion was crucial for reproducing the prickle pattern on ‘Red Queen’ stem around the time $0.0\times T$ (Supplementary Fig. 4d,e).

### Reproduction of prickle patterning on stems of other plant species

**Figure 3:**
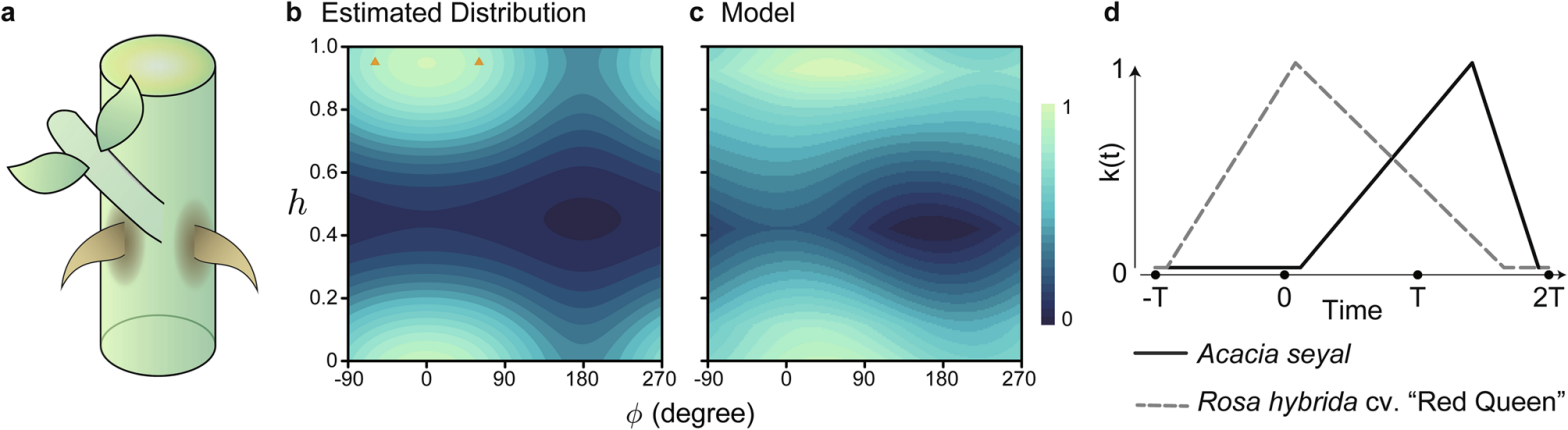
The prickle development model reproduced another prickle pattern on the stem of other plant species. (**a**) The pair prickles associated with leaf base of *A. seyal* (The graphical view is drawn with reference to the book of Adrian et al., pp. 146 and 147^11^). (**b**) The *h*-$\phi$ plane representing the estimated prickle pattern of *A. seyal*; a pair of prickles locates at $h=0.95$ and $\phi =60^{\circ },-60^{\circ }$ (orange triangles). (**c**) Computer simulation of prickle density using a set of optimized parameters at $\alpha =0.044$, $\beta =0.102$, $T_a=0.109$, $T_b=1.419$ and $T_c=1.925$. The Pearson’s correlation to the data of (**b**) is 0.94. (**d**) Plot of the time-dependent production rate *k*(*t*).

We tested whether our model reproduced prickle patterning on stems of other plant species. The prickles of many plants emerge as a pair on either side of the base of a leafstalk. For example, *Rosa hirtula* and *Acacia seyal* have a pair of the prickles at the same height and adjacent to stipules^11^ (Fig. 3a). We found the parameter set reproducing the pair pattern having a peak just below the leaf on $\phi$-*h* plane through the optimization algorithm (Fig. 3b, 3c). The base of leafstalk should inhibit the development of prickles around $\phi =0$ and $h=1$ (or $h=0$). The inhibition can produce the pair pattern from the one peak. The inhibitor release time in the parameter set that reproduced the paired prickle pattern differed from that in the ‘Red queen’ parameter set (Fig. 3d). The curve of the inhibitor distribution in the pair prickle parameter set was flatter than the curve of the distribution in the ‘Red queen’ parameter set (Supplementary Fig. 5). This result suggested that differences in parameters of factor diffusion can account for the diversity in prickle patterning among plant species.

### Distance between prickles

We measured the length $d_{pp}$ between a prickle and the nearest one, and the results indicated that interactions among prickles affected prickle pattern. The $d_{pp}$ of the ‘Red queen’ prickles peaked between 5 mm and 10 mm, and the pairs of prickles closer than 5 mm were rarely observed (Supplementary Fig. 6a). This result suggested that the prickle emergence inhibited the development of other prickles within approximately 5 mm radius, possibly through chemical inhibition or physical factors, such as excluded volume effects. Fused prickles were observed occasionally, indicating imperfectness of mutual exclusion on the stem (Supplementary Fig. 6b,c).

## Discussion

### Relevance of the model to the real plant development

Our model employed a hypothetical diffusible inhibitor signal that inhibits the development of prickles. Instead of the inhibitor, it might be possible to assume the activator in a similar model framework. In trichome development in Arabidopsis, which is likely to be regulated by the conserved gene with prickle development in rose^15^, it is already known that MYB transcription factors acting as negative regulators directly move between cells^18^. Although the inhibitor(s) exerted by leaf primordia has not been identified, the trichome patterning is regulated by microRNA as well as phytohormones^22^, which can be diffusible positional signals. In phyllotaxis, which is one of the most studied topics in plant pattern formation, an abstract model assuming inhibitory field around leaf primordia has been suggested^23^. This inhibitory field was later mapped to the dynamics of the phytohormone auxin and its efflux carrier PIN1^24^. We expect that specific signals for prickle development will similarly be identified and mapped to our abstract model.

Our model reproduced two different prickle patterns. $\alpha$ optimized for ‘Red Queen’ was 6.06 times larger than that for paired pattern represented by *R. hirtula* and *A. seyal*, while $\beta$ was only slightly (1.35 times) larger in ‘Red Queen.’ Although quantitative measurements of shoot apex for these two patterns are currently not available to our knowledge, our results suggest that plastchrone or internode growth is larger in ‘Red Queen’ than the paired pattern. In the paired pattern, $T_a$ was positive, indicating that only primordia below the priming zone inhibit prickle initiation. This suggests that only the adaxial side (i.e., the side facing the shoot apex) of leaf primordia affects prickle initiation. On the adaxial side, specific gene expression occurs leading to boundary and axillary meristem formation^25^. Therefore, it is likely that epidermal differentiation is inhibited in the adaxial side.

### Further explorations of prickle developments and patterns

This study investigated the spatial pattern of mature prickles that were $\ge$ 5 mm in height. Small prickles with height < 5 mm were not measured (Supplementary Fig. 7). These small prickles appeared frequently at the bottom side of *R. hybrida* ‘Red Queen’ stems. A similar phenomenon was reported on the stem of *R. hybrida* ‘Laura’^21^. Several rose species exclusively have small prickles on the stem. The small prickle pattern has not been described, and further studies will investigate this spatial pattern. These data will provide valuable information to refine our current model. Currently, it’s technically challenging to measure the height and angle of small prickles. Automating these measurements using imaging analysis techniques will facilitate the determination of not only small prickles but also mature prickles spatial patterns^26–28^.

When we divided the height from the root to the top leaf into the three layers, unimodal distribution occured in the top layer and bimodal distribution occurred in the two lower layers (Supplementary Fig. 8). There were few mature prickles with angles between $-45^{\circ }< \phi < 0^{\circ }$ in the top layer. This result suggests that growth rates in prickles differ in the $\phi -h$ plane, the parameters in our model changes slightly during rose growth, or else prickles initiate at various stages of shoot development in addition to the priming zone. The time-lapse observation in future studies will provide data for these analyses.

The number of prickles on plant stems significantly vary between species, and this study identified two different patterns. Future studies using our developed method will identify additional prickle patterns in other plant species. We will determine whether modifying the parameter values used in our rose model reproduces the observed differences in prickle patterning in other species. If we can perturb the spiral phyllotaxis by genetically or chemically modifying, such as by mutant and inhibitors, we can also determine whether our model reproduces the perturbed pattern. It will provide insights into the identification of factors behind prickle development.

It is generally accepted that spiny structures on the stem play a key role in defense against herbivore^29,30^. Prickles also function as attachment devices to prevent slipping off of supporting structures. Previous studies explored these two roles by focusing on the shape and physicochemical/biological properties of the spiny structures on the stem^31–33^. Further studies should examine the relationship between those roles and observed prickle patterns.

Humans began cultivating roses several thousand years ago. Rose genetics and biology have been investigated because of its economic value. However, this is the first report to show the prickle spatial pattern with the statistical data and mathematical model. We developed the model based on the spiral phyllotaxis of leaves, which was confirmed to decipher the prickle spatial pattern in other rose species. In our model, we estimated the prickle density distribution, instead of directly calculating the position of each prickle. This approach would be effective to discuss highly variable patterns similar to prickle positions, and to find regularity hidden by the variation. We expect further development of this study for understanding diverse heterogeneous patterns that occur in nature.

## Methods

### Plant materials and measurement

The *R. hybrida* ‘Red Queen’ was purchased from Keisei Rose Nurseries Inc., Chiba, Japan. Plants were cultivated in three pots placed at the open field under natural daylight. We measured prickle and leaf patterns on the lateral shoot terminating with a flower, within a week after blooming (Fig. 1a). The angles and positions of prickles and leaves on shoot were measured using a protractor and calipers. We measured the mature prickles over 5 mm in height. We measured the height (*H*) from the base of the lateral shoot to leaf or prickle and the angular position of leaf or prickle around the stem ($\theta$) (Fig. 1b). We defined $\theta =0$ at the direction from the lateral shoot to the primary shoot. To express the relationship between degree and height as a function, $\theta$ was modified to $\theta _c$ that is cumulative value of $\theta$, where $\theta _c = \theta + 360N$, and *N* is non-negative integers. For leaves, *N* was selected so that $\theta _c$ increases monotonically from the bottom of the shoot to its apex. For prickles, we selected *N* based on the spline curve connecting the leaf position $\theta _c$ drawn on the $\theta _c-H$ plane (solid line in Fig. 1c and Supplementary Fig. 2). The spline curve was expressed as a function of height, *H*, $\hat{\theta }_{sp}(H)$. The relative angular prickle position ($\phi$) was defined as the angle from the spline curve, namely, $\phi _i = \theta _{ci} - \hat{\theta }_{sp} (H_i)$, where *i* is the prickle index of assigned consecutively from bottom to top. *N* of prickle was selected so that $\phi$ of prickle ranged from $-90^{\circ }$ to $270^{\circ }$. We defined *h* to represent the height relative to the internode length, where $h=0$ and $h=1$ correspond to the lower and upper node, respectively. Small prickles with a height of $\le$ 5 mm were not measured. We observed both clockwise and counterclockwise spiral phyllotaxis, but the measurement was performed so that the average of divergence angles $\Delta\theta$ was < $180^{\circ }$. All data was analyzed using R program ver. 3.5.2. This research complies with relevant institutional, national, and international guidelines and legislation.

### Computational modeling

Let $\phi$ be an angle on the circle of the priming zone and set $\phi =0$ for the direction of the *n*-th primordium, without loss of generality. Suppose that the inhibitor can diffuse only on the meristem surface. The concentration of diffusive particles (i.e., inhibitors) produced in a single point and randomly diffused on a 2D plane obeyed the normal distribution at equilibrium state. We assumed that the diffusion rate of diffusion factors were rapid enough to ignore the transition state before equilibrium. Under the assumption, the inhibitor distribution on the meristem was a two-dimensional Gaussian distribution, suggesting that the factors on a circle with a distance *m* from the source followed the von Mises distribution^34^. Thus, the intensity of inhibitor effect exerted by a primordium *i* at a angle of $\phi$ on priming circle with a radius of 1 was approximately proportional to the von Mises distribution: $f_i(\phi ,t) = k(t)\exp (m\cos (\phi -\phi _i))$, where $\phi _i$ is the direction of *i*-th primodium and *k* is a time-dependent parameter. We set $t=0$ at the time when *n*-th primoidium passes the priming zone. The value of *m* should be proportional of the distance between the inhibitor source and the priming circle center. Therefore, we set $m(t)=\max [\alpha t + \beta , 0]$, where $\alpha > 0$ corresponds to the velocity of the constant deviation of leaf primordia from the shoot apex. We assumed the inhibitor secretion was maximized when primordia passed through the vicinity of the priming zone, thereby depending on *t*. The magnitude of secretion, *k*(*t*), was represented as a piecewise linear function:

$$\begin{aligned} k(t)={\left\{ \begin{array}{ll} \frac{t-T_a}{T_b-T_a}, &{}T_a< t< T_b\\ \frac{T_c-t}{T_c-T_b}, &{} T_b \le t < T_c\\ 0, &{} \text {otherwise} \end{array}\right. } \end{aligned}$$

where $T_a< T_b < T_c$, $-T \le T_a$, $0 < T_c \le 2T$, implying that only $n-1$, *n* and $n+1$ primordia contribute to inhibitor distribution on the priming circle. The total inhibitor intensity produced by every primordium at time *t*, $f(\phi )$ can be described as,

$$\begin{aligned} f(\phi ,t)&= {} \sum _i{f_i(\phi ,t)}\\&= {} k(t-T)\exp \left\{ m(t-T)\cos (\phi -\Phi )\right\} \\&\quad +k(t)\exp \left\{ m(t)\cos \phi \right\} \\&\quad +k(t+T)\exp \left\{ m(t+T)\cos (\phi +\Phi )\right\} . \end{aligned}$$

The parameters in this model ($\alpha$, $\beta$, $T_a$, $T_b$, and $T_c$) were optimized to maximize the correlation between actual prickle pattern, $p(\phi ,h)$ and the reciprocal of the amount of inhibitor, $f(\phi ,t)^{-1}$. Real data was used to estimate $p(\phi ,h)$. In our model, the unit of *T* is arbitrary. Without loss of generality, herein we set $T=1$.

The real prickle distribution was based on the observation $f_r(\phi , t)$ was obtained through kernel density estimation using the observed prickle arrangement. We sampled the values of $f(\phi _i, t_j)$ and $f_r(\phi _i, t_j)$, where $\phi _i= 2\pi i/N_d$, $t_j=jT/N_d$ and the division number $N_d =$ 100. The cost function was introduced as the Pearson correlation coefficient for the sampled values of *f* and $f_r$, multiplied by $-1$. Kernel density estimation was performed using the function kde2d in the R MASS package ver 7.3-50. The optimization procedure was performed using the function optim with BFGS method in the R stats package ver 3.6.0^35^.

## Supplementary Information

Supplementary Information.
