# Supplementary material for: Discovery of spatial pattern of prickles on stem of Rosa hybrida ‘Red Queen’ and mathematical model of the pattern
Source: Sci Rep. 2021 Jul 5;11:13857. doi: 10.1038/s41598-021-93133-8 (PMC8257614; doi:10.1038/s41598-021-93133-8)
Supplement: Supplementary file 1 — Supplementary Information. [file 41598_2021_93133_MOESM1_ESM.pdf]

Supplementary Information

Discovery of spatial pattern of prickles on stem of *Rosa hybrida* 'Red Queen' and mathematical model of the pattern

Kazuaki Amikura*, Hiroshi Ito, and Miho S. Kitazawa

*Corresponding author: kazuaki.amikura@gmail.com

a

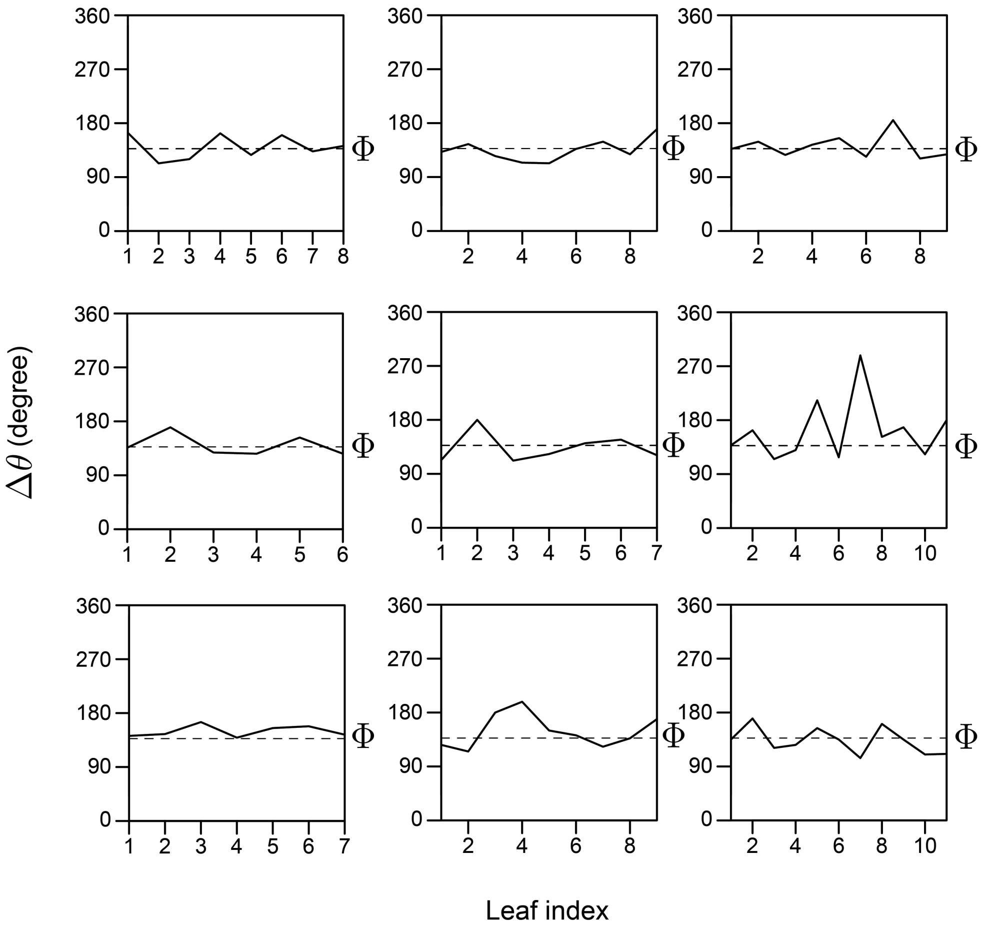

b

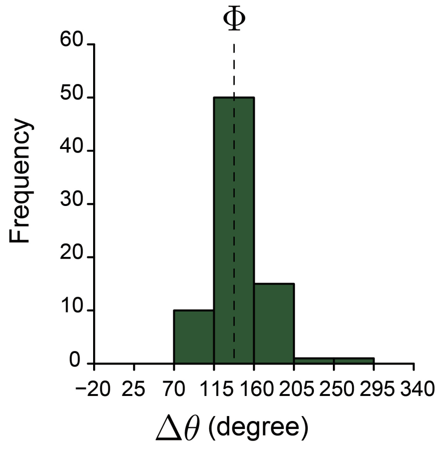

Supplementary Figure 1. Differential values of leaf degrees. a, In order from the top of each stem, the number of leaf index was assigned. $\Delta\theta$ is the difference of the θ_c between the adjacent leaves. The Φ is about 137.5° which in golden angle. b, The histogram represents the $\Delta\theta$ from all samples.

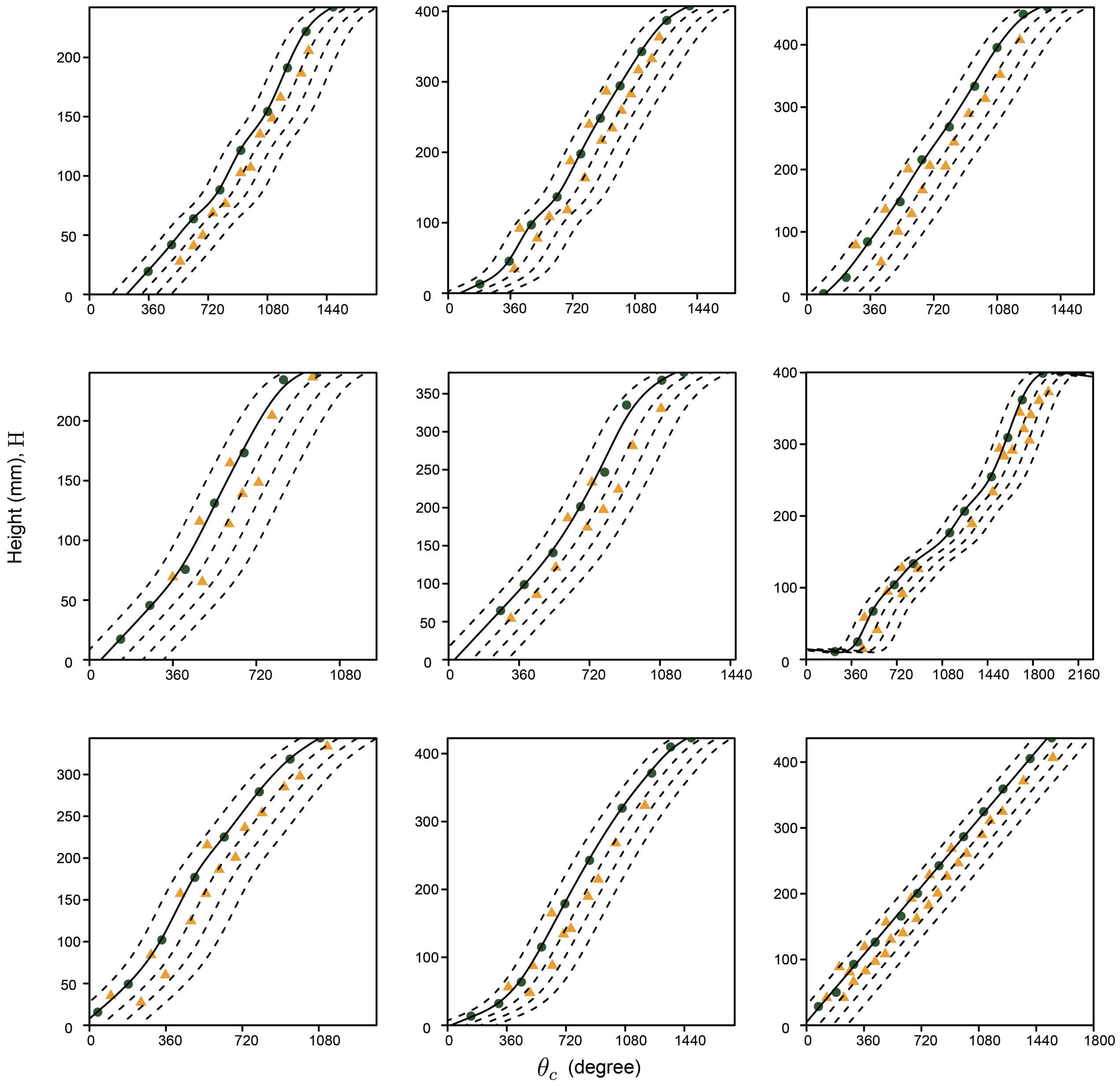

Supplementary Figure 2. The H-θ_c plane of all samples.

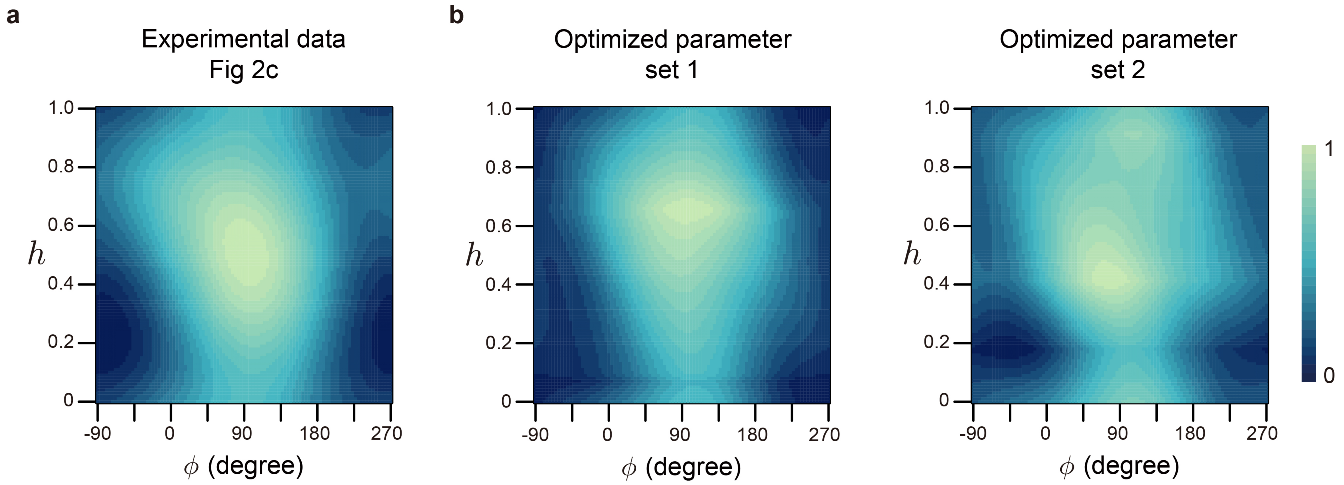

Supplementary Figure 3. Analysis variation of H-ϕ plane using optimized parameter. a, Estimated distribution of prickles on the ϕ-h plane through kernel density estimation for real data (the left panel in Fig 2c). b, Optimized two parameter sets were obtained by maximizing the Pearson's correlation between density from real data and a model. The Pearson's correlation of left panel is the highest, 0.944 (the right panel in Fig 2c). The Pearson's correlation of right panel is 0.940. The optimized parameters are at $\alpha = 0.174$, $\beta = 0.304$, $T_a = -0.57$, $T_b = 0.175$, $T_c = 2.00$.

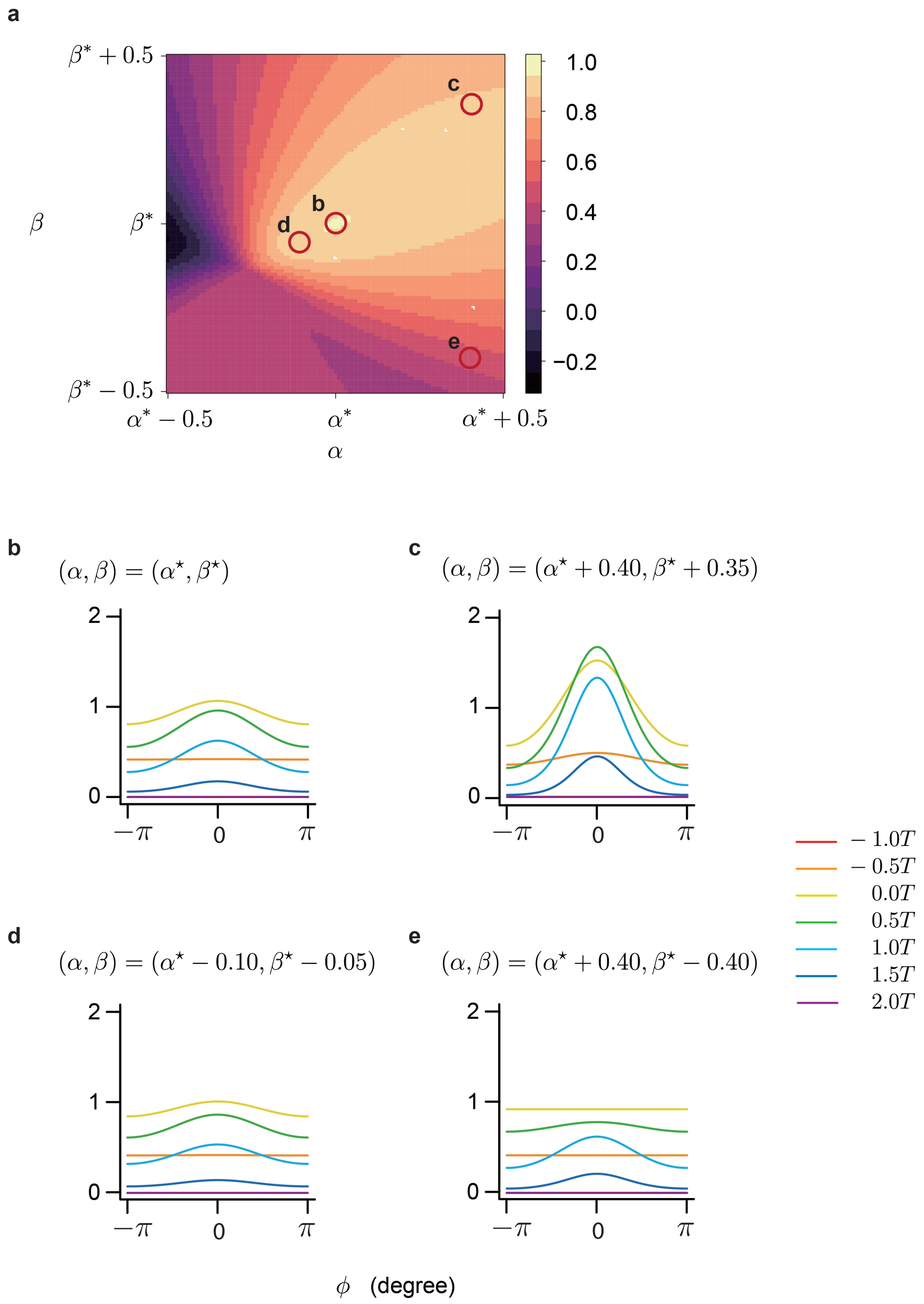

Supplementary Figure 4. Parameter sensitivity. a, The correlation between the real data and the simulation data. The T_a, T_b and T_c is fixed at optimized parameters at Figure 2c(the optimized parameters shown in Fig. 2c). α^* and β^* is 0.267 and 0.139. b-d. Simulation results agree with the real distribution when $f(\phi,t)$ is concave shape at $0.0T$. e. the correlation between simulation and real data is low when $f(\phi,t)$ is flat at $0.0 \times T$.

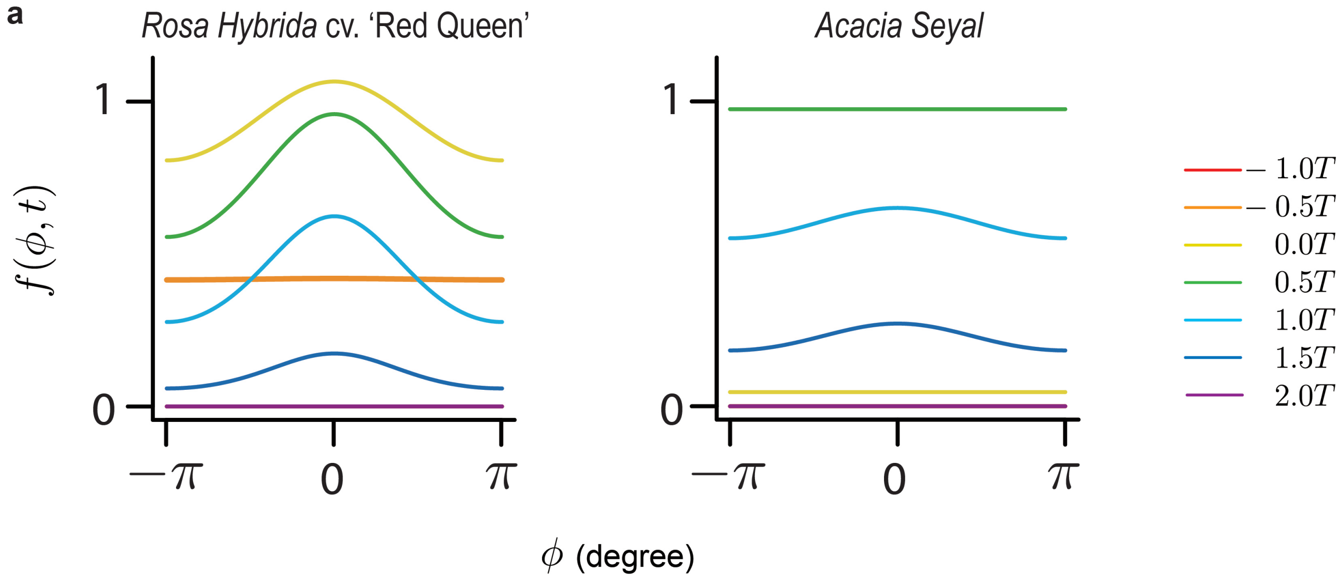

Supplementary Figure 5. $f(\phi,t)$-ϕ graph. a, The graph drawn by the set of parameters of Figure 2c. b, The graph drawn by the set of parameters of Figure 3c.

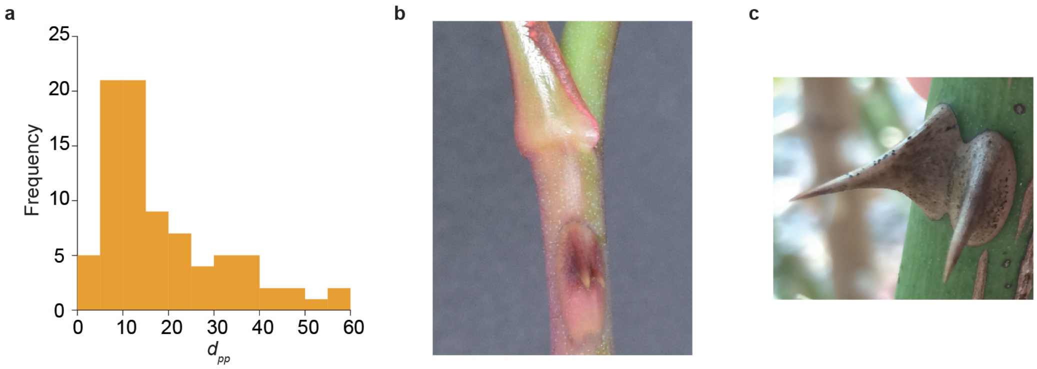

Supplementary Figure 6. Interactions between prickles. a, the distribution of d_{pp}. b, Fused prickle on the stem of *Rosa hybrida* cv. 'Ingrid Bergman'. c, Fused prickle on the stem of *Rosa hybrida* cv. 'Carinella'.

a

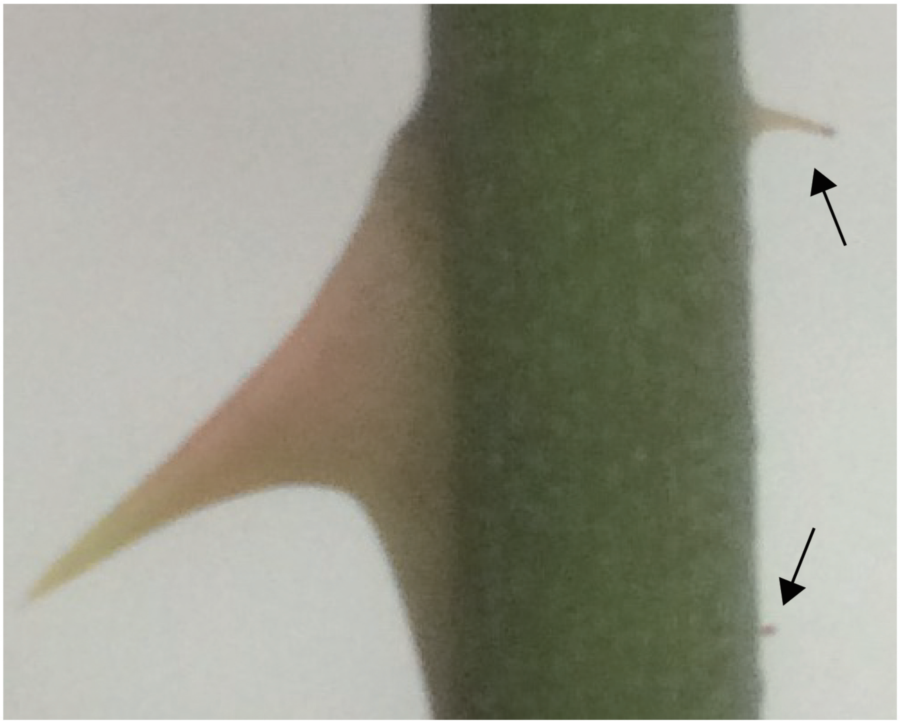

Supplementary Figure 7. Small prickles. a, Large and small prickle on the stem of *Rosa hybrida* cv. ‘Red Queen’. The black arrow is pointing to the small prickle.

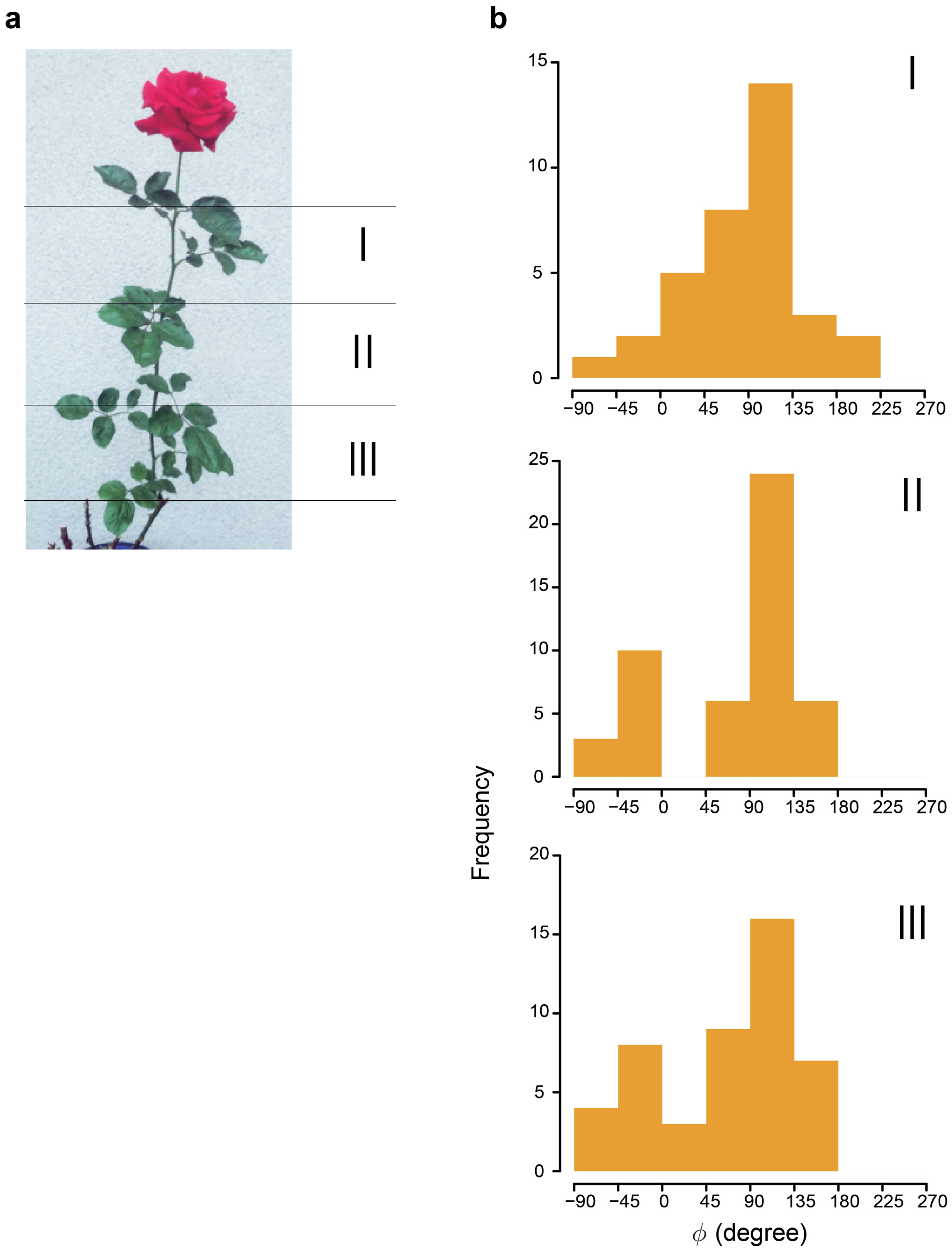

Supplementary Figure 8. Bimodality of the distribution of ϕ. a, The divided area in height dependence. b, The histogram of h in the each area.
